# Interferon inducible pseudouridine modification in human mRNA by quantitative nanopore profiling

**DOI:** 10.1186/s13059-021-02557-y

**Published:** 2021-12-06

**Authors:** Sihao Huang, Wen Zhang, Christopher D. Katanski, Devin Dersh, Qing Dai, Karen Lolans, Jonathan Yewdell, A. Murat Eren, Tao Pan

**Affiliations:** 1 Department of Biochemistry & Molecular Biology, University of Chicago, Chicago, IL 60637 USA; 2 Laboratory of Viral Diseases, National Institute of Allergy and Infectious Diseases, NIH, Bethesda, MD 20892 USA; 3 Department of Chemistry, University of Chicago, Chicago, IL 60637 USA; 4 Department of Medicine, University of Chicago, Chicago, IL 60637 USA

**Keywords:** Pseudouridine, Nanopore sequencing, Interferon, Machine learning

## Abstract

Pseudouridine (Ψ) is an abundant mRNA modification in mammalian transcriptome, but its functions have remained elusive due to the difficulty of transcriptome-wide mapping. We develop a nanopore native RNA sequencing method for quantitative Ψ prediction (NanoPsu) that utilizes native content training, machine learning modeling, and single-read linkage analysis. Biologically, we find interferon inducible Ψ modifications in interferon-stimulated gene transcripts which are consistent with a role of Ψ in enabling efficacy of mRNA vaccines.

**Supplementary Information:**

The online version contains supplementary material available at 10.1186/s13059-021-02557-y.

## Background

Pseudouridine (Ψ) is the second most abundant mRNA modification in the mammalian transcriptome as measured by quantitative mass spectrometry [1] and may exert many cellular functions. For example, Ψ incorporation in synthetic, transfected reporter mRNA increases translation [2] through decreased activation of the RNA-dependent protein kinase (PKR) [3]. The innate immune evading property of Ψ (and its methylated derivative N^1^-methyl-Ψ ) in mRNA is essential to the remarkable immunogenicity of successful COVID-19 mRNA vaccines [4].

Functional exploration and mechanistic investigation of mRNA Ψ modification requires appropriate mapping methods. Illumina sequencing of Ψ in mRNA relies on chemical RNA treatments that induce stop, mutation, or deletion signatures in cDNA synthesis [1, 5–8]. Many computational methods have been developed to map mRNA Ψ sites [9–20]. However, mRNA Ψ mapping is inconsistent among these studies, in part due to the high false positives and negatives generated by the chemical treatments. The read-length limitation of Illumina sequencing also narrows the possibility to examine Ψ usage in mRNA splice isoforms and the linkage of multiple Ψ sites in single molecules.

The emergence of nanopore sequencing enables direct interrogation of RNA modifications [21–23]. Additionally, nanopore sequencing can extend to the full length of the mRNA [24], revealing all modified sites in single RNA isoforms [25]. Both signal strength and dwell time have been used to identify Ψ [26]. Recently, a nanopore direct RNA sequencing method, nanoRMS was developed by Novoa and co-workers that employs characteristic base-calling “error” signatures in the nanopore data for Ψ mapping [27]. NanoRMS identified new Ψ sites in mitochondrial rRNA, small nuclear RNA, small nucleolar RNA, and mRNA under normal and stress conditions in yeast and further, predicted stoichiometry via supervised learning. Although nanoRMS prediction of Ψ site incorporation using a threshold for base mismatch frequency is straightforward, it is unclear whether this approach can be applied to the mammalian transcriptomes, which are much larger than yeast, can contain introns and occur in multiple isoforms. For example, the standard Tombo software for nanopore data analysis is ineffective with spliced reads. Also, even though nanoRMS collects features from single reads, the single read features were averaged before Ψ prediction, erasing single molecule Ψ site incorporation information.

## Results and discussion

**Fig. 1:**
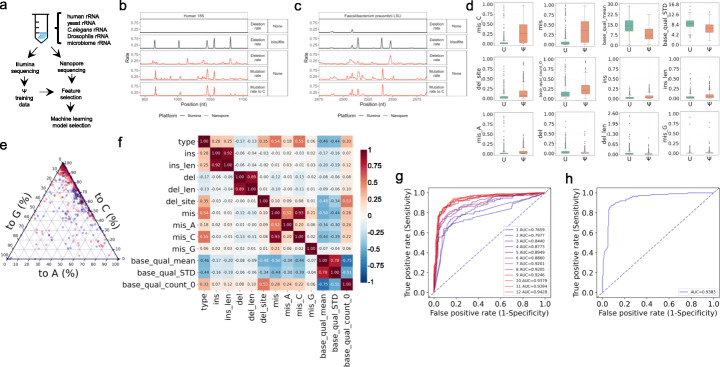
Ψ prediction model training using model organisms and microbiome rRNA Ψ modification. **a** Overview of the experiments to generate the Ψ prediction model by nanopore sequencing. **b** Features of a region in human 18S rRNA from Illumina sequencing and nanopore sequencing. **c** Features of a region in a microbial rRNA from Illumina sequencing and nanopore sequencing. **d** Box and Whisker plots with 1.5 times interquartile range of the 12 feature candidates of U and Ψ sites derived from nanopore sequencing. Ins, insertion rate after the base. Ins_len, insertion length mean. Del, deletion rate after the base. Del_len, deletion length mean. Del_site, deleted site ratio (the site is in a deletion). Mis, overall mismatching ratio. Mis_A, mutation to A ratio. Mis_C, mutation to C ratio. Mis_G, mutation to G ratio. Base_qual_mean, average base quality score. Base_qual_STD, base quality score standard deviation. Base_qual_count_0, ratio of bases with a quality score 0 at a site. **e** Mutation preference for the Ψ sites in all rRNAs in a ternary plot. Red, Ψ sites in model organisms. Blue, Ψ sites in the microbiome.** f** Correlation matrix of modification state (Ψ=1, U=0) and the 12 feature candidates. The value of correlation coefficient is indicated in each box. Same labels as panel **d**. Label type, modification state. **g** ROC (receiver operating characteristic) curves of EXT models with different numbers of features included. The number of features and AUC (aera under curve) values of each model are indicated by the legend. The features are added to the model in the order of their correlation with the modification state indicated in panel **f**. For example, 1 feature means “mis_C”, 2 features means “mis_C” and ”mis”, and so on. **h** ROC curve of the testing set predicted by the optimized EXT model. The AUC value is indicated in the graph

The key to nanopore identification of RNA modification is to generate training data from known modification sites. Modification training data generation, however, requires reads from long transcripts with distinct sequence contexts at the modification site. To maximize our ability to obtain nanopore training data from as many distinct Ψ sites as possible, we generated a mixture of rRNAs from human, yeast, *Caenorhabditis elegans*, Drosophila, and from human fecal bacteria (Fig. 1a). We Illumina sequenced half of the mixture after fragmentation, using the bisulfite reaction [8] to map rRNA Ψ sites, providing a total of 2142 Ψ sites (Additional file 1: Fig. S1a, Additional file 2: Table S1). In Illumina sequencing of the bisulfite method, Ψ sites are found by RT deletions which enable identification and quantitative assessment of closely spaced rRNA Ψ sites; these sites are more difficult to assess using the more commonly used carbodiimide method that identifies Ψ sites by RT stops. Sequencing the remaining sample via direct RNA nanopore sequencing, we found that 640 of these Ψ sites passed our filter of 20 read coverage for further analysis (Additional file 1: Fig. S1b). The lower number of Ψ sites in nanopore sequencing was in part derived from the 3′ bias of the nanopore sequencing library design where all reads start from the 3′ end of the rRNA. These 640 sites were combined with 689 randomly chosen unmodified U sites as the training data set (Additional file 1: Fig. S1c). The modified and unmodified U sites contained 236 of the 256 NN(Ψ/U)NN different 5mer contexts.

We next extracted nanopore signal features suitable for Ψ identification. NanoRMS [27] found that Ψ had negligible effect on nanopore current signals, which means it is hard to directly identify Ψ from the current squiggles like m^6^A [23, 25, 28]. However, distinct features could be found for Ψ identification. For instance, like NanoRMS, we found that apparent mutation to C is a prominent signature for Ψ modification, with apparent deletion also significant for some Ψ sites (Figs. 1b, c). In total, we examined 12 features of base calling errors and found that Ψ sites tend to have lower base quality mean values and standard deviation (Fig. 1d). The ternary plot of mutation signatures confirmed Ψ sites having a strong preference to be read as a C but not A or G (Fig. 1e). The significance of these features was quantified in the correlation matrix of all features and the modification labels (Fig. 1f). We made an extremely randomized trees (EXT) model to carry out Ψ probability prediction for each U site. To decide the combination of features included in the model, we added one feature at a time in the order of their correlation strength with the modification label. This revealed that the performance of Ψ calling maximized when all 12 features were included (Fig. 1g). Using the optimized parameters of our EXT model, its performance was evaluated by the testing set with an area under curve (AUC) of 0.9383 (Fig. 1h, Additional file 1: Fig. S1d). We named our method nanopore investigation of pseudouridines or “NanoPsu.”

Interferons (IFNs), cytokines produced by nearly all cell types during viral and other microbial infections, play crucial roles regulating immune response [29]. mRNA vaccines incorporate Ψ or m^1^Ψ to evade host cell foreign RNA sensing and enhance mRNA translation. Do endogenous mRNAs also use the same strategy through Ψ modification? IFNs can induce the expression of more than a thousand interferon stimulated gene (ISG) transcripts. ISGs include protein kinase R (PKR) which phosphorylates eIF2α to reduce global translation. It is well established that Ψ-modified reporter mRNA activates PKR much less than the same unmodified mRNA and is translated at much higher levels [3]. We therefore hypothesize that ISG transcripts may have elevated levels of Ψ modification to enhance translation in the presence of PKR.

**Fig. 2:**
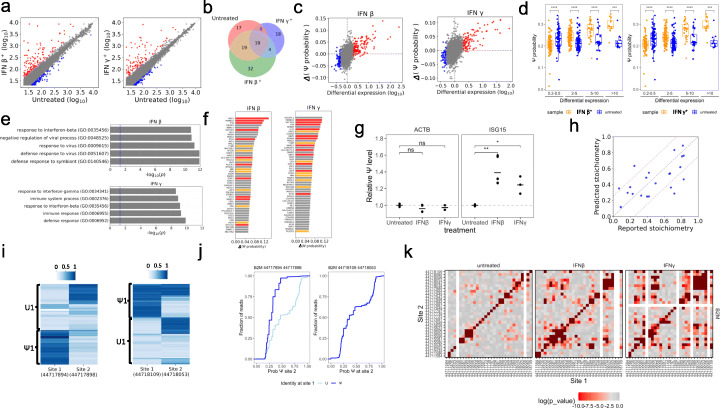
Interferon treatment elicits more Ψ modification in mRNA. **a** Log10 expression levels of genes in untreated sample and IFN β-treated (left) or IFN γ-treated (right) samples. Expression level is calculated as the peak height of the piled reads. Red, genes with an increase of > 2 fold in expression. Blue, genes with a decrease of > 2 fold in expression. **b** Venn diagram of the GO terms of the genes containing the 500 U sites with the highest Ψ probabilities in each sample. **c** Scatter plot showing the mean modification probability change versus log10 expression fold change of each gene between untreated and IFN β-treated (left) or IFN γ-treated (right) sample. Red, genes with an increase of > 2 fold in expression. Blue, genes with a decrease of > 2 fold in expression. **d** Mean Ψ modification probability of genes assigned to groups based on expression fold change between untreated and IFN β-treated (left) or IFN γ-treated (right) samples. ***p<10^-3^, and ****p<10^-4^. **e** GO analysis of the 50 genes with highest mean Ψ probability change between untreated and IFN β-treated (top) or IFN γ-treated (bottom) samples. Blue vertical line indicates *p*=0.05. **f** Mean Ψ probability change of the highest 50 genes between untreated and IFN β-treated (left) or IFN γ-treated (right) samples. Genes with a significant increase in expression levels are marked in red (>10 fold) or orange (5–10 fold). **g** Relative Ψ level of mRNA transcript of ACTB (left panel, data from set 1 and set 2 primers) and ISG15 (right panel, data from set 1, set 2, and set 3 primers) in the untreated and interferon-treated samples measured by RT-qPCR. *p < 0.05; **p < 0.01. **h** Single read prediction results for the partially modified Ψ sites in human rRNA. The stoichiometry predicted by our method is compared with the stoichiometry reported previously by quantitative LC/MS. The correlation coefficient is 0.6566 (Pearson’s r). **i** Clustering heatmap showing the Ψ probability of two pairs of sites in single reads of the B2M transcript in the IFN γ-treated sample. Each row represents a read. Site numbers are defined as the chromosomal locations in the hg38 nomenclature. These two pairs show either negative linkage (left) or no linkage (right). **j** Reads in panel **i** are assigned to “Ψ” and “U” groups based on the posterior probabilities of site 1 in Gaussian mixture model (*k*=2). The cumulative distribution curves of Ψ probabilities of site 2 are drawn for reads in “Ψ” or “U” groups or for all reads. The curves for “Ψ” and “U” groups undergo two sample Kolmogorov-Smirnov test; *p* values are <2.2x10^-16^ (left) and 0.7684 (right). **k** *P* value in the two sample Kolmogorov-Smirnov test for selected pairs of sites in the B2M transcript in the untreated and IFN treated samples

We tested this hypothesis by treating cells with either IFN-γ or IFN-β followed by nanopore sequencing (Additional file 1: Fig. S2a). IFN treatments worked well as determined by upregulation of surface MHC class I (Additional file 1: Fig. S2b). The mRNA expression levels of the biological replicates were highly correlated (Additional file 1: Fig. S2c). For improved coverage, we combined the nanopore data from the biological replicates for downstream analysis (Additional file 1: Fig. S2d). We found strongly upregulated mRNA transcripts upon IFN treatment that belong to the ISG genes with the expected gene ontology of interferon signaling pathway and viral defense (Fig. 2a, Additional file 1: Fig. S3a). These results indicate the feasibility of using nanopore sequencing to study the interferon response transcriptome.

We used the EXT model to predict Ψ modification probabilities in the transcriptome. In total, ~2.6 million U sites were analyzed in each transcriptome (Additional file 1: Fig. S3b). We found a “RAΨU” motif and the previous revealed [30] “GUΨC” motif among top Ψ sites in the untreated sample (Additional file 1: Fig. S3c). The Ψ sites belonging to “median” or higher groups in the previous study [30] showed significantly higher predicted Ψ probabilities than other U sites in the untreated sample (Additional file 1: Fig. S3d), indicating that our method provides valid prediction of Ψ. For the 500 sites with the highest probability of Ψ modification, the three samples shared some but also had distinct sites (Additional file 1: Fig. S3e). However, IFN treated samples had a wider range of GO terms than the untreated sample (Fig. 2b), suggesting that Ψ modification becomes more widespread to transcripts belonging to more diverse cellular processes. Going beyond the top 500 probable Ψ sites, globally the upregulated gene transcripts had higher average modification probabilities for IFN treated vs. untreated samples, with the magnitude of increase strongly correlated with the expression fold change (Figs. 2c, d, Additional file 1: Fig. S3f). Increased Ψ modification probability in a mRNA transcript could be attributed to increased number of Ψ sites and/or increased modification fraction of modified sites. The top 50 genes with highest increase in Ψ modification probability were related to the interferon pathway and anti-viral response (Fig. 2e), they included 88.5% of all genes with >10-fold increase and 60.9% of all genes with >5-fold increase in mRNA expression (Fig. 2f). These results are consistent with increased Ψ modification in the transcriptome upon interferon treatment enhancing ISG function.

We used a RT-qPCR method to validate the increased Ψ level in the ISG transcripts. Our method takes advantage of the standard Ψ detection method using N-cyclohexyl-N′-(2-morpholinoethyl) carbodiimide (CMC). The Ψ-CMC adduct introduces a RT stop in cDNA synthesis which reduces the amount of cDNA product compared to the control reaction without CMC. The differential amount of the cDNA product can then be precisely measured using real-time qPCR (scheme in Additional file 1: Fig. S3g). We first showed that the actin mRNA did not change its abundance nor its Ψ level, making it an appropriate internal control for comparing the Ψ levels of the ISG transcripts (Fig. 2g, Additional file 1: Fig. S3h, left panels). Ψ level increase in the ISG15 mRNA upon interferon treatment was validated upon normalization of its expression level and to the actin mRNA within the same sample (Fig. 2g, Additional file 1: Fig. S3h, right panels).

We performed single-read analysis for quantitative Ψ stoichiometry prediction and investigation of linking modification states of Ψ sites in single molecules of a mRNA transcript. Our training set contained the data points from previously reported [31], 100% modified human rRNA Ψ sites and randomly selected unmodified U sites. We used the same set of features and the same EXT algorithm, while we replaced all the “rate” features (like “mismatch ratio”) with “indicator” features (like “mismatch-or-not”) (Additional file 1: Fig. S4a). We tested the Ψ stoichiometry prediction from single reads on 22 partially modified Ψ sites (5–85%) and found that the predicted stoichiometry matched well with the previous reported stoichiometry obtained by LC/MS [31] (Fig. 2h).

A new aspect of our method is the ability to perform single-read analysis that links occurrence of multiple Ψ sites in individual mRNA transcripts. We examined whether pairs of Ψ site modifications are linked either positively or negatively, meaning whether the modification state of site 2 is affected by the modification state of site 1 and vice versa. We selected 31 positions in the B2M transcript (which encodes the common small subunit of MHC class I molecules) and checked pairwise linkage by two sample Kolmogorov-Smirnov test. In most cases, the maximum distance *D* value from two sample K-S test was small (Additional file 1: Fig. S4b), which is consistent with the presence of Ψ at site 1 being independent of Ψ at site 2, these two sites are not linked. An example of a specific unlinked pair is shown in Fig. 2i, j (right panels). A few pairs of sites had high *D* values, but most of those were immediately adjacent Ψ sites. The pairs of Ψ sites with negative linkage tend to avoid each other in the same mRNA molecule (Additional file 1: Fig. S4c). An example of a specific negatively linked pair is shown in Fig. 2i, j (left panels) where simultaneous Ψ occurrence at both sites is very rare. This result indicates that the modification of Ψ at two sites in single molecule transcripts is negatively related for some and completely independent for others. Upon IFN treatment, the linkage between some sites in the B2M transcript became more prominent (Fig. 2k), suggesting that IFN-induced Ψ installation has stronger co-dependency.

## Conclusions

In summary, we generated a supervised-learning-based protocol to predict Ψ modification in the human transcriptome and analyzed Ψ on single reads which allows for the evaluation of stoichiometry and linkage between distal Ψ sites in the same mRNA molecule. Human genome contains 13 confirmed and putative Ψ installation enzymes [32], suggesting that Ψ installation is a highly robust and dynamic process in human cells. How these enzymes coordinate or antagonize their activities remains to be determined. We found a biological response of Ψ modification change in endogenous mRNA upon IFN treatment which is consistent with Ψ playing a role in IFN signaling pathway and viral defense.

## Methods

### Stool sample collection and total RNA extraction

Stool specimens were self-collected by 1 female volunteer using a commercial “toilet hat” stool specimen collection kit (Fisherbrand Commode Specimen Collection System; Thermo Fisher Scientific, 02-544-208). Specimens were immediately transported to the laboratory (<1h) and thoroughly homogenized. A 100 mg of stool was transferred into a cryovial using a sterile spatula, and 700 μl RNAlater Stabilization solution was added. Specimens were stored at −80°C until extraction.

RNAlater was first removed from stool sample by centrifugation at 17,200 rcf for 10 min at 4°C. Pelleted material was lysed in 400 μL of 0.3M NaOAc/HOAc, 10mM EDTA, and pH 4.8 with an equal volume of acetate-saturated phenol to chloroform pH 4.5 (Invitrogen, AM9722). After addition of 1.0 mm glass lysing beads (Bio-Spec Products, 11079110) in a 1:1 ratio (bead to sample weight), samples were placed in a reciprocating bead beater (Mini-Beadbeater-16, Bio-Spec Products) for two 1-min intervals on maximum intensity. Samples were centrifuged at 17,200 rcf for 15 min at 4°C before re-extraction and isopropanol precipitation of total RNA. Pellets were washed with 75% ethanol before resuspension in an acid-buffered elution buffer (10mM NaOAc, 1mM EDTA, pH 4.8).

### rRNA mixture sample preparation

A mixture of human HEK293T, yeast BY4741 strain, Drosophila S2 cells, and *C. elegans* whole animal and stool microbiome total RNA was made by mixing 1 μg RNA from each model organism sample and 8 μg total RNA from a stool microbiome sample. ZYMO RNA Clean & Concentrator-5 (R1013) kit was used on this mixture to remove all small RNAs <200nt. The final sample was eluted with 20 μl RNase-Free H_2_O. The mixture was split into two halves. One half was used for Illumina sequencing (see below). For nanopore sequencing, the other half was polyadenylated by yeast Poly(A) Polymerase (ThermoFisher 74225Z25KU) by incubation with 0.48 mM ATP, 20 U/μL Poly(A) Polymerase, and 1x Poly(A) Polymerase Reaction Buffer at 37°C for 15 min. The product was size selected using ZYMO RNA Clean & Concentrator-5 (R1013) kit, and RNA molecules >200nt were retained. The sample was eluted with 20 μL RNase-free H_2_O. Then, ~500 ng of this rRNA mixture was used for nanopore direct RNA seq library preparation and nanopore direct RNA sequencing described below.

### rRNA mixture Illumina sequencing and mapping

For Illumina sequencing, bisulfite treatment was performed as described previously [8]. Ψ modification was identified through the deletion at the Ψ site in the sequencing data. Raw reads were demultiplexed via a 4nt barcode on read 2 using je suite [33] with the following parameters: je demultiplex F1=#read1 F2=$read2 BF=$barcode_key BPOS=BOTH BM=READ_2 LEN=6:4 O=$output. Only read 2 from paired-end reads were mapped with bowtie2 (version: bowtie2-2.3.3.1-linux-x86_64) [34] using the following parameters: bowtie2 -x $reference -U $read2 -S $ouput -q -p 10 --local --no-unal. Reads were mapped to either a set of rRNA from model organisms or a set of bacterial rRNA reads: rfam family RF02541 (bacterial large subunit) and RF00177 (bacterial small subunit). SAM files from bacterial rRNAs were processed with a custom python script to count the total number of reads mapping to each sequence. Only sequences with >1000 reads were processed further. Model organism rRNA sequences from human (NCBI: NR_003286.4, NR_003287.4), yeast (RNACentral: URS00005F2C2D_559292, URS000061F377_4932), *C. elegans* (RNACentral: URS00005A42AA_6239, URS00008C9AB9_6239), and Drosophila (RNACentral: URS000030AF9A_7227, URS000008C6A9_7227) to form a reference genome for bowtie mapping. Bowtie2 output “sam” files were converted to sorted bam files with samtools [35]. IGV was used to calculate deletion rates with the following parameters: igvtools [36] count -z 5 -w 1 -e 250 --bases $input $output $reference. Custom python scripts were used to reformat the “wig” file.

### Nanopore direct RNA seq library preparation and sequencing

The library preparation followed the protocol of Direct RNA Sequencing Kit (SQK-RNA002) provided by Oxford Nanopore Technology. Briefly, ~500 ng of Poly(A)^+^ RNA sample was used for each run. Each single run contained one biological replicate of one sample. The RT Adaptor (RTA) was ligated to the 3′ end of Poly(A)^+^ RNA by T4 DNA ligase (NEB M0202S) and then reverse transcribed by SuperScript III Reverse Transcriptase (ThermoFisher 12574018). The RNA was purified by 1.8x RNAClean XP beads (72 μL) (Beckman Coulter A63987) and then the RNA Adaptor (RMX) was ligated to the 3′ end of Poly(A)^+^ RNA using T4 DNA ligase (NEB M0202S) and then the RNA was purified with 1x RNAClean XP beads (40 μL). The sample was eluted with 21 μl Elution Buffer. Then, the sample was loaded onto a R9.4.1 flow cell (FLO-MIN106D) in a MinION sequencer. Each flow cell was sequenced for 72 h.

### Nanopore data pre-processing

All raw fast5 files generated during sequencing were uploaded to Midway2 cluster for the following steps. Reads were base called by guppy base caller (version 3.2.2+9fe0a78) with min_qscore 7. The reads were aligned to by minimap2 (version 2.18-r1015) [37] with parameters -ax splice -uf -k14. The rRNA mixture reads are aligned to the same reference as the rRNA Illumina seq data described above. The human mRNA reads are aligned to the hg38 human genome reference (GRCh38.p13). The mapped reads were piled up to the reference chromosomes by samtools (v1.11). The “error” features were extracted from the mpileup files by customized python scripts (https://github.com/sihaohuanguc/Nanopore_psU).

### Model training

For nanopore seq data of rRNA, all sites mapped to “T” in the reference with >20 coverage made up the data pool. 640 Ψ sites revealed by Illumina sequencing and 689 randomly selected U sites from the data pool made up the model training dataset. The dataset was divided into 60% training set, 20% validation set, and 20% testing set. The Ψ modification prediction models were generated by training set and validated with the validation set by extremely randomized trees (EXT) models with 1–12 features and customized parameters. Then, the models were applied to predict Ψ modification probabilities of the testing set and evaluated by AUC of ROC (receiver operating characteristic) curves derived from the predicted probabilities of the testing set. The final model used EXT algorithm (n_estimators=200, criterion= “gini”, max_depth=None, min_samples_split=2) with 12 features.

### HeLa cell culture and interferon treatment

HeLa cells (ATCC, authenticated and tested for mycoplasma contamination) were cultured in the presence of 500 U/mL human interferon gamma (IFN γ, Peprotech), 500 U/mL human interferon beta (IFN β, Peprotech), or left untreated, with biological duplicates for each. Cells were incubated for 24 h, and an aliquot of each was processed for flow cytometry. Cells were washed into a flow cytometry staining buffer (FBS-containing RPMI and Hanks’ Balanced Salt Solution) containing the anti-pan-MHC-I antibody W6/32 (BioXcell) conjugated with AlexaFluor 647 (Invitrogen). Cells were then washed 3× and analyzed by a Fortessa X-20 (BD Biosciences) to determine upregulation of MHC class I. The rest of the cells were used for RNA extraction via the RNeasy Mini kit (Qiagen) following the manufacturer’s protocol. RNA was eluted in pure water and quantified by Nanodrop (Thermo). PolyA+ RNA from 50 μg total RNA of each sample was extracted by Promega PolyATtract® mRNA Isolation Systems Z5310. Each sample was eluted with 15 μL H_2_O.

### Prediction of Ψ in HeLa samples

The raw data of two replicates for the untreated, IFN γ-treated, and IFN β-treated samples were merged after aligned to the hg38 human genome reference (GRCh38.p13). The merged samples were down sampled so that they have almost the same number of reads and are directly comparable. The Ψ modification probabilities of all sites mapped to “T” in the reference with over 20 coverage were evaluated by the EXT model generated with the rRNA mixture sample. The coverage independence of Ψ probability was examined by down sampling all sites of the samples to similar coverages (expectation = 30) using different random seeds. We found that the change in mean Ψ probability of the transcripts maintained the same after down sampling. The coverage completeness of the transcripts was checked by counting the U sites predicted in the samples [38]. For the untreated sample, the U sites within 5′UTR, CDS, and 3′UTR represented 2.43%, 42.84%, and 54.73% of all U sites, respectively. The gene information was provided by the comprehensive gene annotation file (gencode.v37.annotation.gff3) in the GENCODE database (https://www.gencodegenes.org) [39]. The gene ontology (GO) analysis was performed using the Gene Ontology Resource (http://geneontology.org) [40, 41]. The sequence logo plots were generated by MEME (https://meme-suite.org/meme/tools/meme) [42].

### CMC-mediated RT-qPCR (CRP) validation of Ψ level in mRNA transcripts

#### Primer design

qPCR primers were designed using NCBI Primer-BLAST tool (https://www.ncbi.nlm.nih.gov/tools/primer-blast/). Two to 3 sets of primers were selected to cover the 3′ end, middle, and 5′ end region of the whole transcript. qPCR was performed with TaqMan style fluorescent probes. Probes for each PCR primer pair were designed using IDT PrimerQuest tool (https://www.idtdna.com/pages/tools/primerquest) and examined using NCBI nucleotide BLAST. Primers and probes were purchased from IDT. Actin (NM_001101.5) and ISG15 (NM_005101.4) transcripts were selected for Ψ validation. Below is the list of the sequences of qPCR primers and probes.

ISG15 primer1-Forward: GTGGACAAATGCGACGAACC

ISG15 primer1-Reverse: ATTTCCGGCCCTTGATCCTG

ISG15 probe1: 5′-/56-FAM/TCC TGG TGA/ZEN/GGA ATA ACA AGG GCC/3IABkFQ/-3′

ISG15 primer2-Forward: GCGCAGATCACCCAGAAGAT

ISG15 primer2-Reverse: GTTCGTCGCATTTGTCCACC

ISG15 probe2: 5′-/56-FAM/TTC CAG CAG/ZEN/CGT CTG GCT GT/3IABkFQ/-3′

ISG15 primer3-Forward: CAGCGAACTCATCTTTGCCAG

ISG15 primer3-Reverse: GACACCTGGAATTCGTTGCC

ISG15 probe3: 5′-/56-FAM/TGG GAC CTG/ZEN/ACG GTG AAG ATG C/3IABkFQ/-3′

ACTB primer1-Forward: ACAGGAAGTCCCTTGCCATC

ACTB primer1-Reverse: CAGTGTACAGGTAAGCCCTGG

ACTB probe1:5′-/56-FAM/ACA CGA AAG/ZEN/CAATGCTATCACCTCCC/31ABkFQ/-3′

ACTB primer2-Forward: AGATGTGGATCAGCAAGCAGG

ACTB primer2-Reverse: GGGGGATGCTCGCTCCA

ACTB probe2: 5'-/56-FAM/TCG TCC ACC/ZEN/GCA AAT GCT TCT AGG/31ABkFQ/-3′

### CMC-mediated RT-qPCR (CRP) experiment

CMC [N-cyclohexyl-N′-(2-morpholinoethyl) carbodiimide] treatment was done as previously described [43]. 1.5 μg of untreated, IFNβ-treated, and IFNγ-treated total RNA in 12 μl was mixed with 24 μl TEU buffer (50 mM Tris-HCl (pH 8.3), 4 mM EDTA, 7 M urea) in microcentrifuge tubes. Four microliters of freshly made 1 M CMC (Sigma, C1011) in TEU buffer or 4 μl TEU buffer was added to each sample for +CMC or −CMC treatment, respectively. The sample mixture in 40 μl 0.7× TEU was incubated at 37°C for 1 h. The mixture was diluted to 200 μl with 160 μl of 50 mM KOAc (pH 7) and 200 mM KCl. One microliter of 5 μg/μl glycogen and 550 μl ethanol were added to the mixture to precipitate RNA at −80°C for >2 h. The mixture was then centrifuged at highest speed (17000×*g*) for 30 min. The RNA precipitate was mixed with 500 μl 75% ethanol and kept at −80°C for >2 h followed by centrifugation at 17000×*g* for 30 min. The washing step was repeated once. The RNA precipitate was mixed with 50 μl of 50 mM Na_2_CO_3_ and 2 mM EDTA (pH 10.4) and incubated at 37°C for 6 h to remove CMC-U/CMC-G adducts. The RNA was purified using Zymo RNA Clean and Concentrator column (Zymo, R1014) with in-column DNase treatment by following the manufacturer’s manual. The RNA was eluted in 11 μl sterile H_2_O. The concentration of the ±CMC treated RNA was measured using Nanodrop, and equal amount (~300 ng) of total RNA was used for RT-qPCR experiment.

Eleven microliters of 300 ng ±CMC-treated total RNA from untreated/IFNβ/IFNγ samples were mixed with 1 μl 50 μM 5′T_22_VN (*V*=A,C,G, *N*=A,C,G,T) primer (IDT) and 1 μl 10 mM dNTP mix. The mixtures were incubated at 65°C in thermal cycler for 5 mins followed by incubation at room temperature for 3 min. The PCR tubes were kept on ice until the addition of the SuperScript IV RT mix. 7 μl RT mix was prepared for each sample by combining 4 μl 5× SSIV Buffer, 1 μl 100 mM DTT, 1 μl RNaseOUT RNase inhibitor, and 1 μl SSIV reverse transcriptase. 7 μl RT mix was added to each PCR tube. The tubes were incubated at 55°C in thermal cycler for 1.5 h. The PCR tubes were then incubated at 80°C for 10 min followed by incubation on ice immediately to deactivate RT. 45 μl sterile H_2_O was added to each tube to dilute the RT mixture to 65 μl, and 2 μl was used for qPCR reaction.

qPCR reaction was performed in 10 μl consisting of 5 μl 2× PrimeTime Gene Expression Master Mix (IDT, 1055772), 2 μl RT mix, and 3 μl primer and probe mix. Three microliters of primer and probe mix (1.5 μM each PCR primer and 0.6 μM probe) was first added into each well of 384-well plate or 96-well plate. RT mix of each sample and 2× PrimeTime Gene Expression Master Mix were mixed at 2:5 ratio to make master mix based on the number of qPCR reactions for each sample. Seven microliters of the template and PrimeTime master mix were then added to each well. The plate was spun on a swing bucket plate centrifuge at 3000 RPM for 2 min. qPCR reaction was performed on Bio-Rad CFX384 or CFX96 qPCR machine for 40 cycles. C_q_/C_T_ values were obtained for follow-up data analysis.

Relative Ψ levels for ISG15 transcript was calculated using ACTB-1 as internal reference. First, we obtained ΔCq(-) = Cq(ISG15,-CMC) - Cq(ACTB,-CMC), and ΔCq(+) = Cq(ISG15,+CMC) - Cq(ACTB,+CMC); then, we obtained ΔΔCq(ISG15) = ΔCq(+) - ΔCq(-). The relative Ψ level is represented as 2^ ΔΔCq(ISG15).

### Single read Ψ prediction model training

The 100% modified human rRNA sites were reported in a previously work measured by quantitative LC/MS [31]. A basic assumption was that all reads in our human rRNA sample would have Ψ at the reported 100% modified sites and U at the reported completely unmodified sites. The dataset for training contained 25 100% Ψ sites with 49,437 data points and 26 randomly selected U sites with 50,922 data points. The dataset was divided into 60% training set, 20% validation set, and 20% testing set. Features were extracted from each base in each read. The features describing the ratios in bulk prediction model were replaced with features indicating the mismatching and indel states of the base. The Ψ modification prediction models were generated by training set and validated with the validation set using the EXT algorithm (n_estimators=200, criterion= “gini”, max_depth=None, min_samples_split=2) with 10 features, which are insertion_ot_not, insertion_length, deletion_or_not, deletion_length, deleted_site_or_not, mismatch_or_not, mutate_to_A, mutate_to_C, mutate_to_G, base quality score. The AUC value for the prediction of testing set was 0.8269. To further evaluate the model, Ψ modification probabilities of data points from 22 previously reported [31], partially modified human rRNA Ψ sites (modification fraction from 5 to 85%) were predicted. The base was viewed as Ψ when the probability was larger than 0.5 and as U when the probability was less than 0.5. The stoichiometry of each site was calculated as the number of predicted Ψ bases divided by the coverage of the site.

### Single read Ψ analysis in HeLa samples

The Ψ probabilities of all U residues in selected genes were predicted with the protocol above. To investigate the linkage of multiple Ψ on single reads, each read was indexed so that the U data points with the same read index were from the same read. Ψ probabilities of residues of a certain site were fitted by Gaussian mixture model (GMM) with 2 components. The sites with abs(μ_1_-μ_2_)>0.5 and λ_1_ and λ_2_>0.05 were selected for following analysis. When doing pair wise linkage analysis, the reads were assigned into “Ψ” and “U” groups when it had >95% posterior probability for one population in the GMM for site 1. To evaluate whether there was a difference in the Ψ probabilities distribution of site 2 upon the presence or absence of Ψ at site 1, two sample Kolmogorov-Smirnov test was performed on the Ψ probabilities cumulative distribution curves of site 2 in the “Ψ” and “U” groups with an output of the maximum distance *D* value and *p* value. The R library to do a two-sample Kolmogorov-Smirnov test was from GitHub (https://rdrr.io/github/happyrabbit/DataScienceR/man/pairwise_ks_test.html).

## Supplementary Information

**Additional file 1.** Supplementary figures. Supplementary figures S1-S4.

**Additional file 2.** Ψ sites by Illumina seq. It’s the list of Ψ sites in the rRNA mixture sample identified by our Illumina sequencing experiment.

**Additional file 3.** Review history.

## Data Availability

The datasets generated and analyzed during the current study are available in the NCBI GEO database under the accession GSE180656 [44]. The scripts for “NanoPsu” Ψ prediction package are available on GitHub [45] (https://github.com/sihaohuanguc/Nanopore_psU, GNU General Public License v2.0) or Zenodo [46] (DOI: 10.5281/zenodo.5711328).
