# Supplementary material for: Interferon inducible pseudouridine modification in human mRNA by quantitative nanopore profiling
Source: Genome Biol. 2021 Dec 6;22:330. doi: 10.1186/s13059-021-02557-y (PMC8646010; doi:10.1186/s13059-021-02557-y)
Supplement: Supplementary file 1 — Additional file 1. Supplementary figures. Supplementary figures S1-S4. [file 13059_2021_2557_MOESM1_ESM.pptx]

## Slide 1
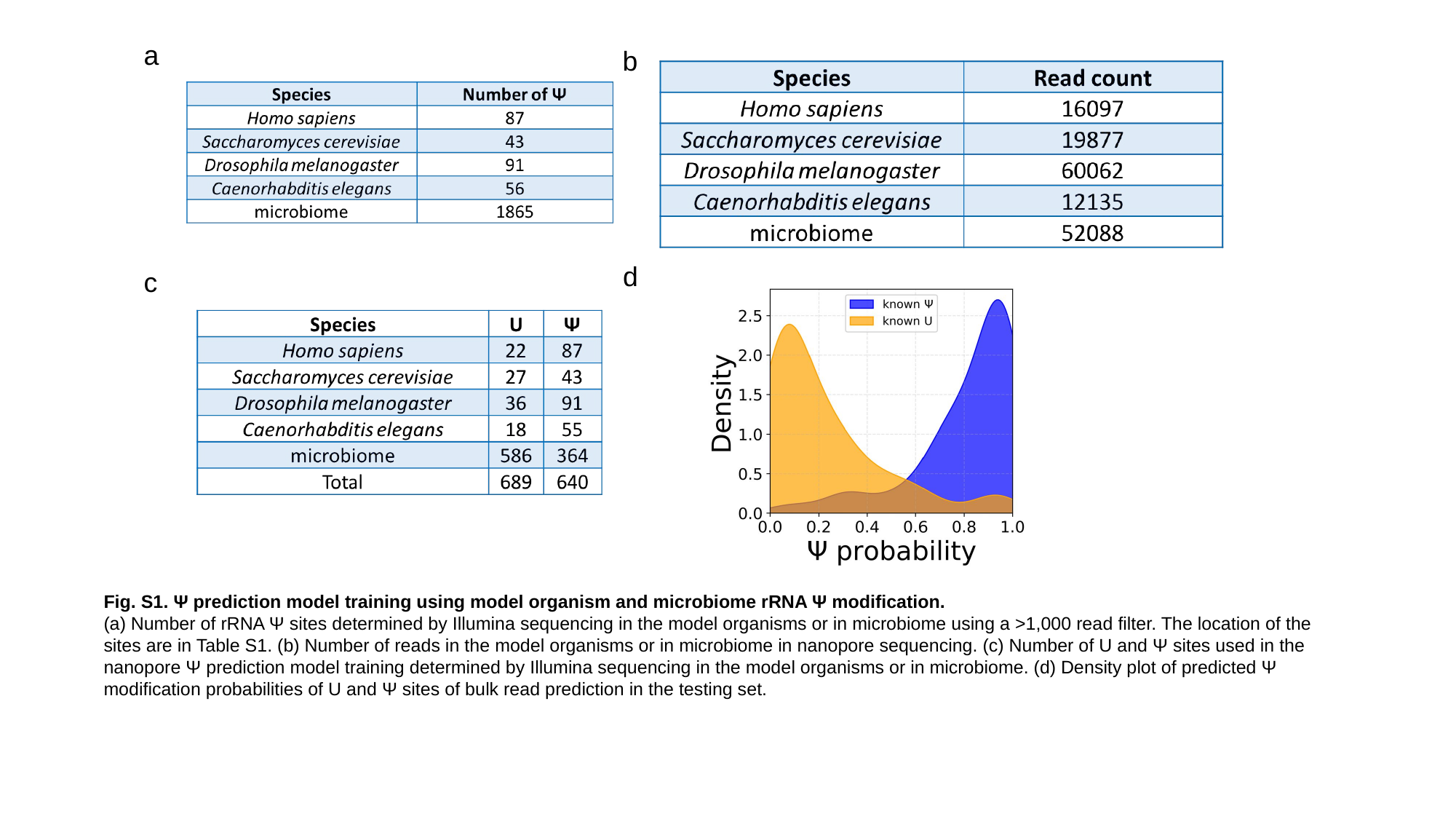

a
b
d
c
Fig. S1. Ψ prediction model training using model organism and microbiome rRNA Ψ modification.
(a) Number of rRNA Ψ sites determined by Illumina sequencing in the model organisms or in microbiome using a >1,000 read filter. The location of the sites are in Table S1. (b) Number of reads in the model organisms or in microbiome in nanopore sequencing. (c) Number of U and Ψ sites used in the nanopore Ψ prediction model training determined by Illumina sequencing in the model organisms or in microbiome. (d) Density plot of predicted Ψ modification probabilities of U and Ψ sites of bulk read prediction in the testing set.

## Slide 2
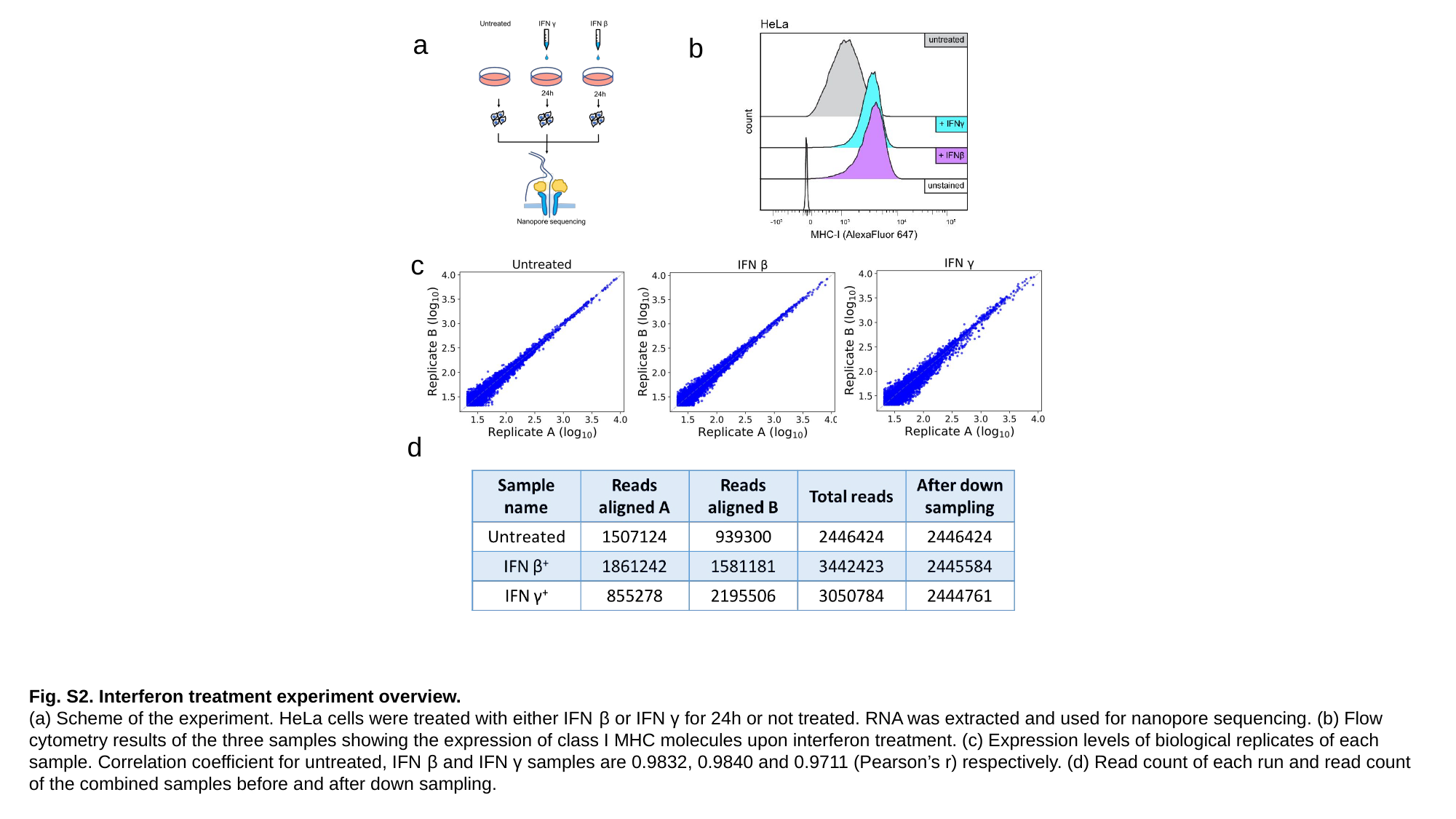

a
b
c
d
Fig. S2. Interferon treatment experiment overview.
(a) Scheme of the experiment. HeLa cells were treated with either IFN β or IFN γ for 24h or not treated. RNA was extracted and used for nanopore sequencing. (b) Flow cytometry results of the three samples showing the expression of class I MHC molecules upon interferon treatment. (c) Expression levels of biological replicates of each sample. Correlation coefficient for untreated, IFN β and IFN γ samples are 0.9832, 0.9840 and 0.9711 (Pearson’s r) respectively. (d) Read count of each run and read count of the combined samples before and after down sampling.

## Slide 3
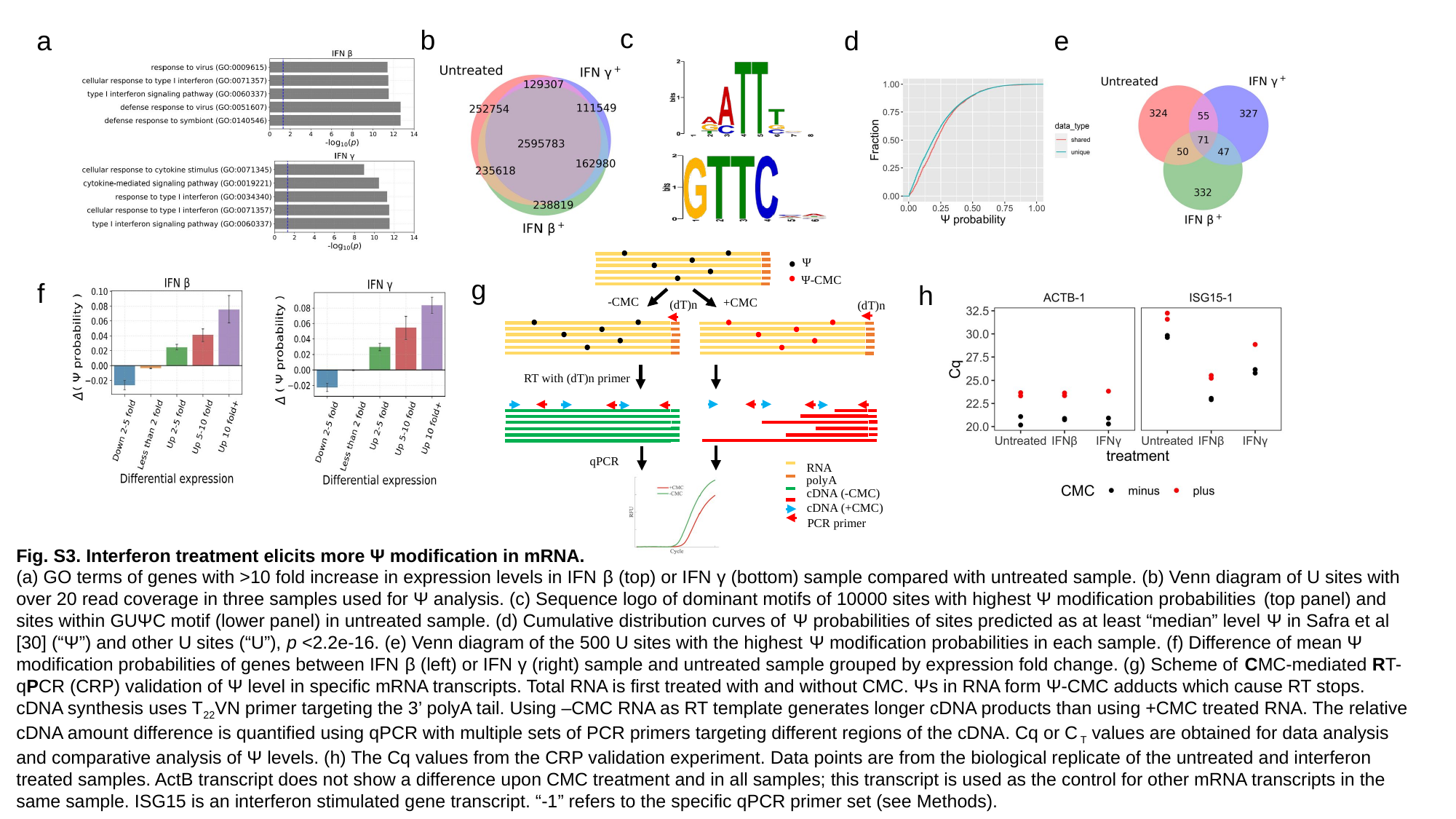

c
b
d
e
a
Ψ
Ψ-CMC
-CMC
+CMC
(dT)n
(dT)n
RT with (dT)n primer
qPCR
RNA
polyA
cDNA (-CMC)
cDNA (+CMC)
PCR primer
g
Δ
f
Δ
h
Fig. S3. Interferon treatment elicits more Ψ modification in mRNA.
(a) GO terms of genes with >10 fold increase in expression levels in IFN β (top) or IFN γ (bottom) sample compared with untreated sample. (b) Venn diagram of U sites with over 20 read coverage in three samples used for Ψ analysis. (c) Sequence logo of dominant motifs of 10000 sites with highest Ψ modification probabilities (top panel) and sites within GUΨC motif (lower panel) in untreated sample. (d) Cumulative distribution curves of Ψ probabilities of sites predicted as at least “median” level Ψ in Safra et al [30] (“Ψ”) and other U sites (“U”), p <2.2e-16. (e) Venn diagram of the 500 U sites with the highest Ψ modification probabilities in each sample. (f) Difference of mean Ψ modification probabilities of genes between IFN β (left) or IFN γ (right) sample and untreated sample grouped by expression fold change. (g) Scheme of CMC-mediated RT-qPCR (CRP) validation of Ψ level in specific mRNA transcripts. Total RNA is first treated with and without CMC. Ψs in RNA form Ψ-CMC adducts which cause RT stops. cDNA synthesis uses T22VN primer targeting the 3’ polyA tail. Using –CMC RNA as RT template generates longer cDNA products than using +CMC treated RNA. The relative cDNA amount difference is quantified using qPCR with multiple sets of PCR primers targeting different regions of the cDNA. Cq or CT values are obtained for data analysis and comparative analysis of Ψ levels. (h) The Cq values from the CRP validation experiment. Data points are from the biological replicate of the untreated and interferon treated samples. ActB transcript does not show a difference upon CMC treatment and in all samples; this transcript is used as the control for other mRNA transcripts in the same sample. ISG15 is an interferon stimulated gene transcript. “-1” refers to the specific qPCR primer set (see Methods).

## Slide 4
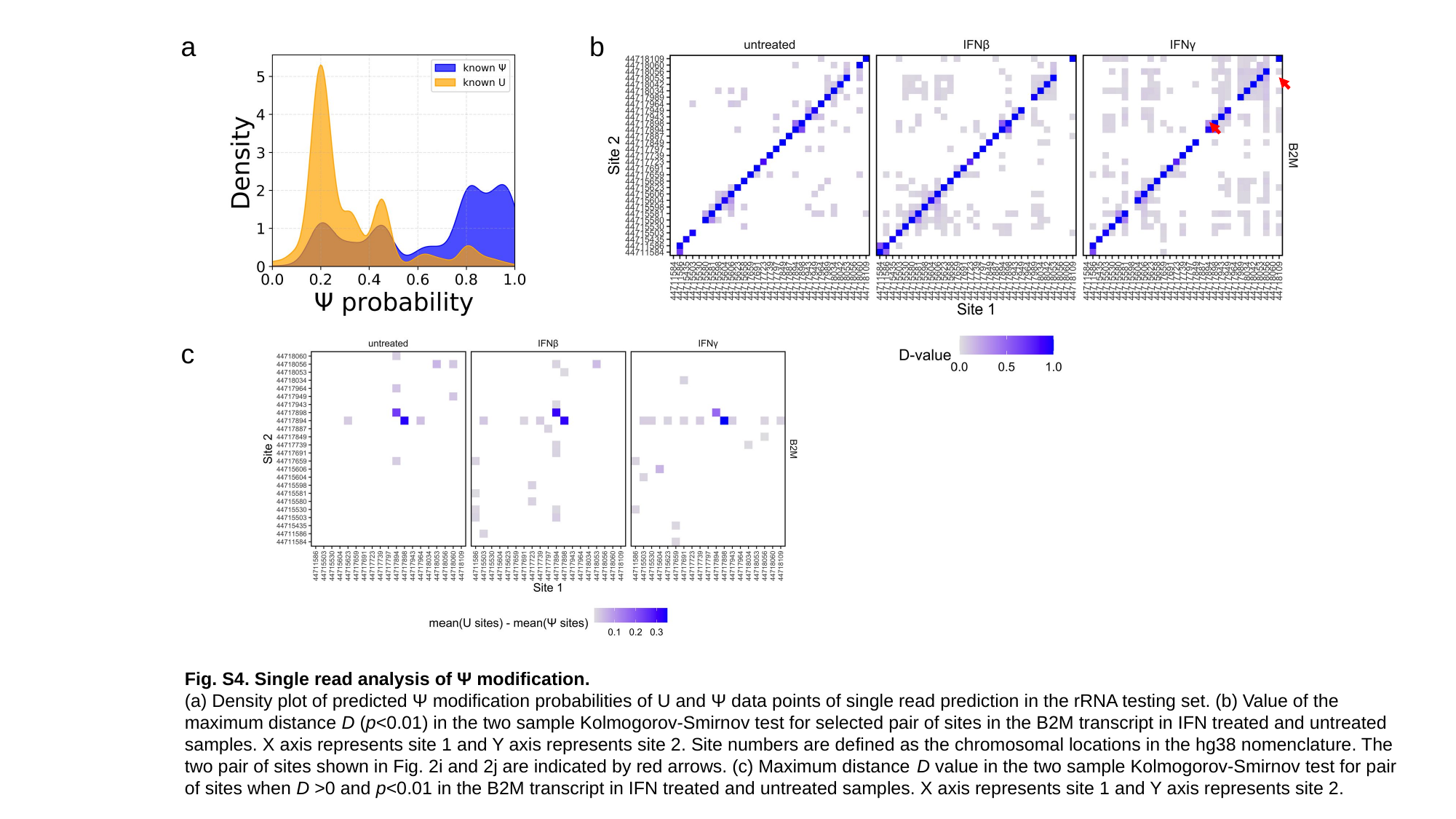

a
b
c
Fig. S4. Single read analysis of Ψ modification.
(a) Density plot of predicted Ψ modification probabilities of U and Ψ data points of single read prediction in the rRNA testing set. (b) Value of the maximum distance D (p<0.01) in the two sample Kolmogorov-Smirnov test for selected pair of sites in the B2M transcript in IFN treated and untreated samples. X axis represents site 1 and Y axis represents site 2. Site numbers are defined as the chromosomal locations in the hg38 nomenclature. The two pair of sites shown in Fig. 2i and 2j are indicated by red arrows. (c) Maximum distance D value in the two sample Kolmogorov-Smirnov test for pair of sites when D >0 and p<0.01 in the B2M transcript in IFN treated and untreated samples. X axis represents site 1 and Y axis represents site 2.
