# Supplementary material for: Interferon inducible pseudouridine modification in human mRNA by quantitative nanopore profiling
Source: Genome Biol. 2021 Dec 6;22:330. doi: 10.1186/s13059-021-02557-y (PMC8646010; doi:10.1186/s13059-021-02557-y)
Supplement: Supplementary file 3 — Additional file 3. Review history. [file 13059_2021_2557_MOESM3_ESM.docx]

Review History

**First round of review**

**Reviewer 1**

**Were you able to assess all statistics in the manuscript, including the appropriateness of statistical tests used?**

No, I do not feel adequately qualified to assess the statistics.

**Comments to author:**

In the current study, the authors describe a nanopore direct RNA-seq method to identify and quantify Ψ in the human transcriptome. The authors successfully used this method to identify Ψ level changes in interferon-inducible gene transcripts.

This manuscript is one of the first nano-pore sequencing studies revealing Ψ sites, especially on the endogenous genes. This work represents a significant advance over previously published studies on Ψ-seq.

the major conclusion is adequately by the data, However, this reviewer suggests the authors consider the following points:

1. The authors used rRNA Ψ sites in microbiomes making machine learning approaches. Can the authors comment on the discrepancies of bisulfite-seq identified >2000 sites and the nanopore-identified 600 sites? Why use bisulfite but not CMC-seq on rRNA?

2. Can the authors experimentally prove the increased Ψ sites on the top targets identified in nanopore seq?

**Reviewer 2**

**Were you able to assess all statistics in the manuscript, including the appropriateness of statistical tests used?**

Yes, and I have assessed the statistics in my report.

**Comments to author:**

This paper was interesting to read and I think would be of general interest to the readers of Genome Biology if some concerns can be addressed, some of which I view as critical.

Critical issues:

1. The authors' coverage of the relevant literature around pseudouridine detection in RNA could be much better. For instance, there are other tools aimed at pseudouridine detection in RNA including "Porpoise" (https://doi.org/10.1093/bib/bbab245) and "Penguin" (https://doi.org/10.1101/2021.03.31.437901). The former paper (https://doi.org/10.1093/bib/bbab245) has a table (Table 1) with 10 published approaches to identifying pseudoU sites in mRNA, none of which are referenced here. Also, please site computational tools. Je suite: https://doi.org/10.1186/s12859-016-1284-2, Minimap2: https://doi.org/10.1093/bioinformatics/bty191, MEME: https://meme-suite.org/meme/doc/cite.html, etc.

2. This paper has limited value unless accompanied by a usable computational method i.e. a software tool. The github repository "https://github.com/sihaohuanguc/20210715_Nanopore_psU" does not exist. The code at "https://github.com/ckatanski/nanopore_pseudoU_2021" has no usage information and does not appear to be a working tool or pipeline for detecting pseudoU.

3. What implementations of the ML models (RF, EXT, KNN, SVM) were used? What parameters were used? These should be stated and cited. Also - typically when doing model selection there should be a training set, a validation set, and a set on which to test the final model. Model selection, i.e. the part where you've decided the EXT model yields the highest accuracy, would be carried out with the training and validation sets, followed by final assessment using the held-out test set. Here it appears there is only training and validation with at 60/40 split and the 40% set is doing double duty as for both model/parameter selection and the final characterisation of accuracy. If this is indeed what is happening this isn't ideal and the basis for this should be discussed.

Additional issues that should be addressed in a resubmission:

4. Is it possible to seperate expression level and the amount of pseudoU modification? I think it would be helpful to assess the potential for confounding here. In other words, reassure me that you don't see more pseudoU just becasue you see more transcripts.

5. The authors establish that multiple pseudoU sites in the same mRNA molecule are rare - I would be intereted to know whether there is an actual depletion of co-occuring sites or if the rarity is consistent with a stochastic model.

6. What does the 3' bias profile look like from nanopore direct RNA sequencing? Put another way, how often is a complete mRNA molecule sequenced?

**Authors Response**

**Point-by-point responses to the reviewers’ comments:**

Reviewer #1:

In the current study, the authors describe a nanopore direct RNA-seq method to identify and quantify Ψ in the human transcriptome. The authors successfully used this method to identify Ψ level changes in interferon-inducible gene transcripts.

This manuscript is one of the first nano-pore sequencing studies revealing Ψ sites, especially on the endogenous genes. This work represents a significant advance over previously published studies on Ψ-seq.

*Response: We thank the reviewer very much for the encouraging comments.*

the major conclusion is adequately by the data, However, this reviewer suggests the authors consider the following points:

1. The authors used rRNA Ψ sites in microbiomes making machine learning approaches. Can the authors comment on the discrepancies of bisulfite-seq identified >2000 sites and the nanopore-identified 600 sites? Why use bisulfite but not CMC-seq on rRNA?

*Response: (i) The lower number of Ψ sites in nanopore sequencing was derived in part from the 3’ bias of the nanopore data where all reads start from the 3’ end of the rRNA (after the polyA addition in the nanopore library building protocol). In contrast, the Illumina sequencing sample was first fragmented before adapter ligation so the full rRNA was covered more evenly. (ii) In Illumina sequencing of the bisulfite method, Ψ sites are found by deletions which enables identification and quantitative assessment of closely spaced rRNA Ψ sites; these sites are more difficult to assess using the carbodiimide method that identifies Ψ sites by stops.*

*We added a brief description on both points on p. 4, second paragraph.*

2. Can the authors experimentally prove the increased Ψ sites on the top targets identified in nanopore seq?

*Response: Our nanopore result shows increased Ψ levels in interferon stimulated gene transcripts. To validate this, we worked out a RT-qPCR method that takes advantage of the standard Ψ detection method using CMC. The Ψ-CMC adduct introduces a RT stop in cDNA synthesis which reduces the amount of cDNA product compared to the control reaction without CMC. The differential amount of the cDNA product can then be precisely measured using real-time qPCR. We first showed that the actin mRNA did not change its abundance nor its Ψ level, making it an appropriate internal control for comparing the Ψ levels of the ISG transcripts. Ψ level increase in the ISG15 mRNA upon interferon treatment was determined upon normalization of its expression level and to the actin mRNA within the same sample.*

*These results are shown in the new Figs. 2h, S3g, S3h, and the accommodating description is on p. 7, second paragraph, and the method description on pages 15-17.*

Reviewer #2:

This paper was interesting to read and I think would be of general interest to the readers of Genome Biology if some concerns can be addressed, some of which I view as critical.

*Response: We thank the reviewer very much for the encouraging comments.*

Critical issues:

1. The authors' coverage of the relevant literature around pseudouridine detection in RNA could be much better. For instance, there are other tools aimed at pseudouridine detection in RNA including "Porpoise" (https://doi.org/10.1093/bib/bbab245) and "Penguin" (https://doi.org/10.1101/2021.03.31.437901). The former paper (https://doi.org/10.1093/bib/bbab245) has a table (Table 1) with 10 published approaches to identifying pseudoU sites in mRNA, none of which are referenced here. Also, please site computational tools. Je suite: https://doi.org/10.1186/s12859-016-1284-2, Minimap2: https://doi.org/10.1093/bioinformatics/bty191, MEME: https://meme-suite.org/meme/doc/cite.html, etc.

*Response: We now cite all papers on pseudoU detection from the Illumina sequencing data suggested by the reviewer with a brief mention (references 9-20, p. 3, second paragraph). We also added the references for Je suite (reference 33), minimap2 (reference 37) and MEME (reference 42), as well as references for other computational tools used here (references 34-36, 38-41).*

2. This paper has limited value unless accompanied by a usable computational method i.e. a software tool. The github repository "https://github.com/sihaohuanguc/20210715_Nanopore_psU" does not exist. The code at "https://github.com/ckatanski/nanopore_pseudoU_2021" has no usage information and does not appear to be a working tool or pipeline for detecting pseudoU.

*Response: We thank the reviewer for this suggestion. We have made a python package publicly available on github (https://github.com/sihaohuanguc/Nanopore_psU), and provide the link on p. 20, availability of data and materials.*

*We also provide a reviewer token for the deposited nanopore data of GSE180656: urexsgwcblitzct*

3. What implementations of the ML models (RF, EXT, KNN, SVM) were used? What parameters were used? These should be stated and cited. Also - typically when doing model selection there should be a training set, a validation set, and a set on which to test the final model. Model selection, i.e. the part where you've decided the EXT model yields the highest accuracy, would be carried out with the training and validation sets, followed by final assessment using the held-out test set. Here it appears there is only training and validation with at 60/40 split and the 40% set is doing double duty as for both model/parameter selection and the final characterisation of accuracy. If this is indeed what is happening this isn't ideal and the basis for this should be discussed.

*Response: As suggested, we re-split the data into training, validation and testing sets (60%-20%-20%) and trained and optimized the models. We decided to only focus on the EXT algorithm and its optimized parameters in the revision. The final model is essentially the same as the one in the original manuscript. We updated all figures using the revised model.*

*We provide a brief description about this on p. 5, second paragraph, and in the method section, p. 13, second paragraph, and p. 18, first paragraph.*

Additional issues that should be addressed in a resubmission:

4. Is it possible to seperate expression level and the amount of pseudoU modification? I think it would be helpful to assess the potential for confounding here. In other words, reassure me that you don't see more pseudoU just becasue you see more transcripts.

*Response: In principle, our pseudoU prediction program is already normalized to the coverage, so theoretically the expression level should not affect the result. As a test, we used similar numbers of randomly selected reads (expectation = 30 in our case) of each site in untreated and interferon treated samples and repeated the pseudoU probability calculation. For example, for the ADAR gene we randomly down-sampled the untreated and IFN gamma treated samples to the same level 100 times with different random seeds, and run the pseudoU probability calculation. The psU probability increased every time within a narrow distribution (Figure).*

*We added a brief description about this on p. 14, second paragraph.*

5. The authors establish that multiple pseudoU sites in the same mRNA molecule are rare - I would be intereted to know whether there is an actual depletion of co-occuring sites or if the rarity is consistent with a stochastic model.

*Response: Our single read analysis shown here is for a single ISG transcript (B2M), and found that coordinated sites are rare in this transcript. There are 13 pseudoU installation enzymes in the human genome, at this time none of these is well characterized regarding how they modify mRNA. Therefore, we are unable to conclude whether the pseudoU modification in B2M represents active depletion or stochastic events.*

*We added a brief description on this on p. 9, first paragraph.*

6. What does the 3' bias profile look like from nanopore direct RNA sequencing? Put another way, how often is a complete mRNA molecule sequenced?

*Response: As expected, the data has a 3’ bias because of the nanopore sequencing design that starts from the 3’ polyA tail. We calculated the U residue coverage of 5’UTR, CDS and 3’UTR in our data: for the untreated sample, the coverage is 2.43% 5’ UTR, 42.84% CDS, and 54.73% 5’UTR. Given that an average human mRNA length is ~2.7 kb, we more likely get full-length transcripts for short genes.*

*We added this result on p. 14, second paragraph.*

**Second round of review**

**Reviewer 2**

Thank you for careful consideration in addressing the reviewer comments. I think this has been substantially improved and believe it is now acceptable and will contribute substantialy to future studies involving pseudo-U.
